# La importancia de la correlación clínico- epidemiológica en el diagnóstico temprano de la histoplasmosis: reporte de dos casos clínicos en Popayán, Colombia

**DOI:** 10.7705/biomedica.6782

**Published:** 2023-08-31

**Authors:** Jorge Andrés Potosí, Yina Marcela Gutiérrez, Fabiola Eugenia González

**Affiliations:** 1 Laboratorio de Microbiología y Parasitología, Facultad Ciencias de la Salud, Universidad del Cauca, Popayán, Colombia Universidad del Cauca Facultad Ciencias de la Salud Universidad del Cauca Popayán Colombia

**Keywords:** histoplasmosis, diagnóstico, HIV, síndrome de inmunodeficiencia adquirida, salud pública, Histoplasmosis, diagnosis, HIV, acquired immunodeficiency syndrome, public health

## Abstract

La histoplasmosis es una micosis endémica en Colombia. Se presentan dos casos del departamento del Cauca, para mostrar el impacto clínico que conlleva un retraso en su diagnóstico y tratamiento. Se obtuvo el consentimiento informado para revisar las historias clínicas de los pacientes y publicar los casos.

El primer caso se trata de un paciente con infección por el virus de inmunodeficiencia humana (*Human Immunodeficiency Virus,* HIV), quien presentaba lesiones cutáneas generalizadas atribuidas inicialmente al virus del herpes; *post mortem y* mediante el cultivo para hongos de muestras de las lesiones dérmicas, se confirmó el diagnóstico de histoplasmosis. El segundo caso es un paciente inmunocompetente con sintomatología pulmonar, a quien se le diagnosticó tuberculosis clínicamente y se le instauró tratamiento; sin embargo, ante la nula mejoría y teniendo en cuenta el antecedente de ingreso a una cueva de murciélagos, se enfocó como una posible histoplasmosis pulmonar y se obtuvo mejoría con el tratamiento. Se revisó la literatura sobre las pruebas de laboratorio y los datos epidemiológicos de histoplasmosis que deben considerar los profesionales de la salud. Se concluyó que las instituciones de salud deben disponer de pruebas rápidas (por ejemplo, antigénicas) para el diagnóstico y tratamiento adecuado de esta micosis, además de adoptar los correctivos necesarios para minimizar la exposición a *Histoplasma*.

## Abstract (en)

Histoplasmosis is an endemic mycosis in Colombia. Here we present two cases in the Cauca department, to indicate the clinical impact of histoplasmosis delayed diagnosis and treatment when its epidemiology is unknown. Informed consent was requested to review patients’ medical records and case report publication.

The first case was a patient diagnosed with human immunodeficiency virus and generalized presence of skin lesions. Initially, these lesions were diagnosed as herpes, but a postmortem diagnosis confirmed histoplasmosis through fungal cultures of tissues from the skin lesions. The second case is an immunocompetent patient with pulmonary symptoms diagnosed and treated for tuberculosis. However, given the lack of improvement and considering the bat cave entrance history, the patient was treated for possible pulmonary histoplasmosis with an adequate response.

We made a review of laboratory tests and histoplasmosis epidemiological data relevant to health professionals. We concluded that health institutions must provide rapid tests, such as antigen ones, to adequately diagnose and treat this mycosis; and also take corrective measures to minimize exposure to *Histoplasma*.

## Caso uno

Se trata de un hombre de 39 años, procedente del área urbana de Popayán, con infección por HIV detectado tres meses antes y un cuadro clínico de 15 días de evolución caracterizado por la aparición de lesiones cutáneas pleomorfas en diferentes estadios, localizadas en los miembros superiores y en el cuero cabelludo, con posterior diseminación al tronco y a los miembros inferiores. Además, presentaba un síndrome constitucional, marcha inestable y tos de resolución espontánea, sin adenomegalias. Durante la valoración física de ingreso, se encontró hipotenso, bradicárdico y afebril.

Se hizo el diagnóstico clínico de herpes cutáneo generalizado y se manejó con aciclovir. Los resultados del hemograma evidenciaron pancitopenia, valores de deshidrogenasa del ácido láctico (LDH) de 1.011 Ul/L y un recuento de 274 linfocitos CD4+ por µl. Otros exámenes de laboratorio resultaron negativos para patógenos oportunistas: marcadores serológicos de hepatitis B como inmunoglobulinas anticuerpos generales (anti-HBs) y contra el antígeno de superficie (HBsAg) y de la nucleocápisde (anti-HBc); búsqueda de anticuerpos contra hepatitis C (anti-VHC) y treponema (VDRL)-para diagnóstico de sífilis; tinción de Giemsa en lavado broncoalveolar para determinar pneumocistosis, examen coprológico y tinción de Gram en muestra de orina sin centrifugar. Los hallazgos de la radiografía de tórax fueron normales.

Dada la escasa efectividad terapéutica, se sospechó histoplasmosis diseminada. Se inició tratamiento empírico con deoxicolato de anfotericina B por vía intravenosa, en dosis diaria de 0,7 mg/kg, y se solicitó tinción de Giemsa y cultivo para hongos de una muestra tomada por biopsia de las lesiones cutáneas. No se observaron estructuras micóticas en el examen microscópico.

A pesar del tratamiento antimicótico, la salud del paciente continúo deteriorándose. Las citopenias empeoraron, y requirió transfusión de dos unidades de glóbulos rojos y cuatro de plaquetas.

Al sexto día, en la radiografía de tórax se observó engrosamiento peribronquial con infiltrados micronodulares. El paciente presentó convulsiones tonicoclónicas generalizadas, se tornó agresivo y se negó a recibir el tratamiento médico durante dos días. Al noveno día de hospitalización, sufrió un paro cardiorrespiratorio y falleció.

**Figura 1:**
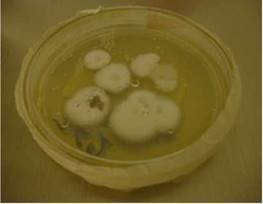
Cultivo de *Histoplasma capsulatum* en medio agar Sabouraud-dextrosa, a una temperatura de 28°C

A los 28 días de incubación del cultivo para hongos, se obtuvo el aislamiento de un moho algodonoso y blanco. El hongo fue teñido con azul de lactofenol y, por microscopía, se observaron hifas tabicadas, delgadas y hialinas. Se hizo un montaje para microcultivo en la búsqueda de estructuras esporuladas que permitieran su identificación y, luego de 10 días, se reportó el aislamiento de *Histoplasma capsulatum* (figuras 1 y 2).

**Figura 2:**
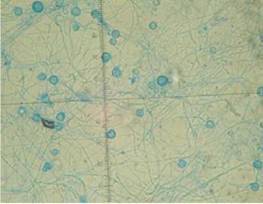
Macroconidias tuberculadas de *Histoplasma capsulatum*

## Caso dos

Se trata de un hombre de 47 años, proveniente del área rural de Timbío (Cauca), agricultor de profesión, que ingresó por un cuadro clínico de 15 días de evolución consistente en tos seca, disnea, cefalea, astenia, adinamia, osteomialgias, fiebre y pérdida de 6 kg de peso, aproximadamente.

En el examen físico se encontró taquicárdico, febril, con 86 % de saturación arterial de oxígeno, leves tirajes intercostales, estertores crepitantes de predominio derecho y ninguna alteración neurológica. Se le practicó una prueba rápida para HIV con resultado negativo, el hemograma y la creatinina se encontraron dentro de límites normales y la proteína C reactiva estaba elevada (15 mg/l).

**Figura 3:**
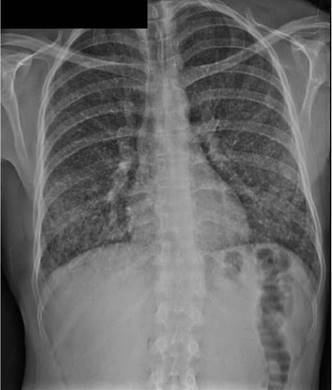
Radiografía de tórax del paciente del segundo caso. Se reportan infiltrados miliares difusos.

En la radiografía inicial de tórax se evidenciaron infiltrados miliares difusos (figura 3). Ante estos hallazgos, se sospechó un cuadro de tuberculosis pulmonar y se ordenó baciloscopia seriada del lavado broncoalveolar. No se encontraron bacilos ácidoalcohol resistentes. El cultivo para micobacterias y la prueba de reacción en cadena de la polimerasa, realizada con el equipo GeneXpert, fueron negativos.

En interconsulta con el especialista en Medicina Interna, el paciente refirió que un mes antes del inicio de los síntomas ingresó por primera vez a una cueva llena de murciélagos para recoger guano, acompañado por otras dos personas, quienes presentaron sintomatología similar, aunque mejoraron espontáneamente. Con los datos anteriores, se planteó la posibilidad diagnóstica de histoplasmosis pulmonar. Se solicitó una prueba con hidróxido de potasio y cultivo para hongos de la secreción obtenida por lavado broncoalveolar, cuyos resultados no se encontraron en la historia clínica.

En búsqueda de compromiso multiorgánico, se ordenaron exámenes para evaluar los niveles de fosfatasa alcalina, transaminasas, bilirrubina total y directa, y deshidrogenasa láctica. Todos los valores se encontraron dentro de los parámetros normales.

Se inició un tratamiento empírico, con deoxicolato de anfotericina B por vía intravenosa, en dosis diaria de 0,7 mg/kg, antes de administrar hidrocortisona. A los 15 días de hospitalización, el paciente presentaba una evolución clínica favorable y se dio de alta con formulación de itraconazol durante seis meses y seguimiento médico ambulatorio.

Al revisar nuevamente la historia clínica, se encontró que el paciente regresó al mes para la consulta de control, señaló que se encontraba en buen estado de salud y no asistió a más consultas. En la visita domiciliaria se detectó, como factor de riesgo epidemiológico, que el paciente tenía galpones para la cría de pollos, por lo cual se le hicieron las respectivas recomendaciones según los *Centers for Disease Control and Prevention* (CDC).

Los aspectos clínicos y epidemiológicos del caso, así como la mejoría clínica obtenida con el tratamiento y el control sin evidencia micológica ni inmunológica de actividad, permitieron hacer el diagnóstico de histoplasmosis pulmonar epidémica, según las definiciones de consenso del Grupo de Enfermedades Infecciosas de la *European Organisation for Research and Treatment of Cancer* y el estudio de Micosis ^1^.

### Consideraciones éticas

Se solicitó consentimiento informado al segundo paciente para la presentación de su enfermedad como caso clínico. Debido al desenlace del paciente uno, se contactó a un familiar quien proporcionó el consentimiento informado para su publicación como caso clínico. Estos dos casos hacen parte del macroproyecto de investigación “Perfil clínico y epidemiológico de las micosis pulmonares en sintomáticos respiratorios de algunas instituciones prestadoras de servicios de salud del Cauca, Colombia”, el cual contó con el aval del comité de ética institucional.

## Discusión

Se presentan dos casos clínicos de pacientes atendidos en centros hospitalarios de Popayán, por su importancia clínica y epidemiológica. En el primer paciente, su estado de inmunosupresión grave por la infección con HIV, con un recuento de 274 linfocitos CD4+ por µl, permitió la colonización oportunista de *H. capsulatum y* desarrolló histoplasmosis diseminada con manifestaciones cutáneas. Estas lesiones posiblemente tenían una baja carga del hongo, ya que este no se observó en el examen microscópico directo. El crecimiento del hongo fue muy lento en los medios de cultivo y se requirió un microcultivo para su identificación final. El proceso de laboratorio convencional fue muy demorado, ya que la micosis se confirmó después de la muerte del paciente, a pesar del tratamiento antimicótico empírico que se le estaba suministrando.

Es de aclarar que no se practicó la prueba inmunológica del antígeno de *Histoplasma* en orina, como indica el protocolo de manejo, ya que en el centro hospitalario donde se encontraba recluido el paciente no estaba disponible.

En el segundo caso, se sospechó inicialmente tuberculosis dada la sintomatología y los resultados de imagenología. Las fallas en la anamnesis inicial no permitieron hacer el diagnóstico presuntivo de esta micosis, pero en una segunda valoración, se sospechó histoplasmosis pulmonar epidémica por el antecedente de haber ingresado a una cueva de murciélagos. Por ser un paciente sin ningún tipo de inmunocompromiso y por el tratamiento empírico antimicótico administrado, se pudo tratar la enfermedad.

La histoplasmosis es una infección endémica en Colombia, que afecta especialmente a personas con riesgo clínico y epidemiológico. Los profesionales de la salud deben hacer una anamnesis detallada que les permita aproximarse a este diagnóstico clínico presuntivo y confirmarlo con las herramientas de apoyo diagnóstico (métodos micológicos convencionales y pruebas inmunológicas para la detección de anticuerpos y de antígenos de *H. capsulatum*).

La histoplasmosis se considera una micosis endémica en países tropicales y subtropicales ^2^. Es causada por el hongo dimorfo dependiente de temperatura *H. capsulatum*, cuyas variedades filogenéticas fueron estudiadas inicialmente por Kasuga *et al*. (2003), quienes identificaron ocho clados: 1) norteamericana clase 1, clado 1; 2) norteamericana clase 2, clado 2; 3) latinoamericana grupo A, clado 3; 4) latinoamericana grupo B, clado 4; 5) australiana, clado 5; 6) holandesa, clado 6; 7) euroasiática, clado 7 y 8) africana, clado 8 (anteriormente variedad *duboisii*) ^3^.

*Histoplasma capsulatum* puede encontrarse en la tierra contaminada con heces de aves y murciélagos, con altas concentraciones de nitrógeno y fosfatos, a una temperatura entre los 22 y los 29 °C, y una humedad relativa entre el 67 y el 87 %. Este hongo es dependiente de la temperatura, debido a que entre los 20 y los 25 °C (temperatura ambiente) se desarrolla la forma saprófita (moho), con formación de macroconidias tuberculadas que varían entre 8 y 15 pm de diámetro, y microconidias o esporas de 2 a 5 pm de diámetro. Dado el tamaño de estas últimas, de inhalarse pueden viajar hasta los alvéolos del huésped; la transición de *H. capsulatum* a una levadura ovoide, de 2 a 5 pm de diámetro, es inducida principalmente por cambios de temperatura mayores de 30 °C ^4^.

Esta micosis está limitada a ciertas regiones endémicas, como el centro- este de los Estados Unidos, donde se calcula que del 80 al 90 % de la población ha sido infectada por *H. capsulatum,* con una incidencia de 1.000 casos por año. En el 2020, se estimaron entre 6.710 y 15.657 los casos de histoplasmosis en pacientes con HIV, con una mortalidad aproximada del 39 % ^5^^-^^7^. Otras zonas endémicas importantes se localizan en Suramérica, Centroamérica y las Antillas. También, se ha descrito la enfermedad en Asia, África, Australia y Oceania, principalmente en zonas de clima templado, subtropical o tropical húmedo ^2^^,^^7^^,^^8^. Sin embargo, los brotes autóctonos en latitudes extremas, como en Canadá y la Patagonia argentina, sugieren una mayor propagación de *H. capsulatum* en el medio ambiente ^9^.

Esta enfermedad cobró importancia para la salud pública por el incremento de su incidencia, especialmente en su forma diseminada progresiva, con el advenimiento de la pandemia por el HIV ^5^^,^^10^^-^^12^. En 1987, los CDC de Estados Unidos determinaron que la histoplasmosis diseminada es una entidad definitoria del síndrome de inmunodeficiencia adquirida (sida) ^13^.

En Colombia, la histoplasmosis no es una enfermedad de notificación obligatoria, pero sí de notificación pasiva. El laboratorio diligencia una encuesta suministrada por el Instituto Nacional de Salud y envía el aislamiento de la cepa con las medidas de bioseguridad correspondientes. El último dato publicado de la Encuesta Nacional de Histoplasmosis en Colombia entre los años 1997 y 2008, reportó 432 casos: 77 % presentados por hombres y 70,5 % con coinfección por HIV. Para el departamento del Cauca, el registro fue del 0,69 % ^14^. Los resultados de la encuesta no reflejan el comportamiento verdadero de la enfermedad, ya que hay un subregistro en aquellos lugares que carecen de un adecuado sistema de vigilancia en salud pública o donde no se reportan los datos.

En el departamento del Cauca, se ha visto un incremento de población vulnerable, como son los pacientes infectados por el HIV: para el año 2022 y según el Sistema Nacional de Vigilancia en Salud Pública (SIVIGILA), la incidencia epidemiológica de VIH/sida en esta región fue de 120 casos ^15^.

Entre las actividades de riesgo epidemiológico para el ser humano tanto en el área rural como en la urbana, están: tareas de limpieza de corrales de aves, áticos y graneros, uso de abonos orgánicos (gallinaza, pollinaza, guano), demolición de edificios, tala de árboles, ingreso a cuevas con murciélagos, actividades de remoción de suelos y limpieza de puentes y alcantarillas. Usualmente, en estos lugares el hongo encuentra las condiciones microambientales que le permiten su reproducción y supervivencia ^5^^,^^7^.

Hay que tener en cuenta la asociación entre brotes de histoplasmosis y perturbaciones ambientales, particularmente en presencia de excrementos de pájaros o murciélagos, ya que las grandes concentraciones de esporas pueden causar la infección ^5^. Este fue la circunstancia de la exposición del segundo paciente y de dos de sus compañeros, quienes también presentaron una sintomatología respiratoria, que se resolvió sin complicaciones.

Se han reportado brotes de esta micosis en Estados Unidos, México y República Dominicana ^5^^,^^16^^,^^17^. En Colombia, también han ocurrido brotes de histoplasmosis en la zona andina. Los pacientes tenían como antecedentes: visitas a cuevas, remoción de tierra de un árbol hueco y de cal agrícola contaminada con excrementos de aves, demolición de casas viejas y uso de tierra abonada con gallinaza y tala de cafetales ^18^. En el 2002, se publicó un brote de histoplasmosis en Medellín, en una familia cuya fuente de infección fue la manipulación de tierra enriquecida con gallinaza ^19^.

En el 2007, Ramírez *et al.* llevaron a cabo un estudio en la zona urbana de Popayán, en el cual observaron que los murciélagos estaban formando sus colonias en los cielos rasos de las casas del sector histórico ^20^. Por lo anterior, durante la consulta médica es importante interrogar al paciente sobre su contacto con posibles focos de infección con esta micosis.

Los aerosoles originados en el suelo contaminado facilitan el ingreso por inhalación de las esporas de *H. capsulatum* en las vías aéreas, donde pueden ser atrapadas por el aparato mucociliar; las que alcanzan a evadir este obstáculo pueden producir una infección pulmonar ^4^. El reconocimiento inmunitario innato de *H. capsulatum* por macrófagos y células dendríticas, es fundamental para la producción temprana de citocinas y quimiocinas, y para el proceso de fagocitosis. Estas células proteínas son necesarias para promover la diferenciación y el reclutamiento de linfocitos T ayudadores (Th1); la falla en generar una respuesta de tipo Th1 resulta en un colapso de la inmunidad ^11^.

En pacientes inmunocompetentes, como el segundo, que viven en zonas endémicas y que pueden inhalar una gran cantidad de esporas, la sintomatología de histoplasmosis generalmente se presenta de una a tres semanas después de la exposición, y puede incluir fiebre, escalofríos, tos seca, dolor torácico, mialgias y cefalea ^21^.

La histoplasmosis tiene dos formas de manifestarse, tanto clínica como radiológicamente. La primera es como una enfermedad pulmonar aguda o epidémica que se acompaña de lesiones neumónicas segmentarias, a menudo grandes, que tienden a curar y se designan como lesiones tempranas. La segunda forma suele presentarse como una complicación de la primera; es una enfermedad crónica caracterizada por cavitación persistente y aumento de la fibrosis pulmonar, y a menudo, por insuficiencia pulmonar progresiva; esta forma se puede confundir con tuberculosis pulmonar ^6^^,^^22^^,^^23^.

En pacientes inmunocomprometidos, como el del caso uno, la histoplasmosis se puede presentar en la forma diseminada progresiva, con síntomas constitucionales y respiratorios, acompañada de signos como hepatoesplenomegalia, linfoadenopatías y lesiones cutáneas ^21^. Los exámenes de laboratorio pueden mostrar anemia, leucopenia, trombocitopenia y valores muy elevados de LDH (superiores a 1.000 ng/ml) y ferritina (mayores de 10.000 ng/ml), que en un contexto clínico adecuado pueden sugerir el diagnóstico de la forma diseminada ^23^^,^^24^. Muchos de estos parámetros clínicos y paraclínicos los presentó el paciente con HIV que aún no estaba en estadio de sida

Según Wheat *et al.*, desde 1990, la sola presencia de fiebre en un paciente con un recuento de linfocitos CD4+ inferior a 150 por pl, acompañada o no de síntomas constitucionales, tos, visceromegalias y lesiones mucocutáneas, debe sugerir la posibilidad de histoplasmosis ^6^.

En la piel, la histoplasmosis primaria se puede presentar por implantación traumática, pero, debido a la poca cantidad de inóculo, es extremadamente rara en pacientes inmunocompetentes y se trata eficazmente con triazoles ^25^^-^^27^.

Según la literatura científica, entre las enfermedades cutáneas más frecuentes en pacientes infectados por el HIV está la histoplasmosis. Se pueden presentar cuando los linfocitos CD4+ se encuentran en cifras inferiores a 250 por µl ^28^. En el caso del paciente número uno, su recuento de CD4+ estaba en 274 por µl. En Latinoamérica, se ha reportado compromiso cutáneo por histoplasmosis en el 38 al 85 % de los pacientes con sida. Las lesiones son polimorfas, como placas con costras o sin ellas, pústulas, nódulos, lesiones semejantes a las del molusco contagioso, erupciones acneiformes o pápulas eritematosas ^29^.

Las pruebas más sensibles para un diagnóstico temprano de histoplasmosis en un paciente con lesiones cutáneas son las tinciones de Giemsa o Wright. Se deben recolectar muestras por escarificación o tejido por biopsia. La observación microscópica de estas muestras permite visualizar las blastoconidias intracelulares de 2 a 5 pm de diámetro, localizadas en el citoplasma de los neutrófilos y de las células fagocíticas del sistema inmunológico. La sensibilidad de estas tinciones es del 25 al 80 % y su especificidad hasta del 90 %, pero los resultados pueden depender de la carga del hongo en la muestra clínica y la experiencia del observador ^30^. Con tinciones histopatológicas para hongos como la de plata metenamina, o de Grocott-Gomori, se observan blastoconidias pequeñas, de color marrón oscuro por la precipitación de los iones de plata ^31^.

Por otro lado, el hallazgo de *H. capsulatum* en extendidos de sangre periférica es poco sensible, pero es simple, rápido y específico en casos diseminados ^32^. Las tinciones deben ir acompañadas del cultivo para hongos de muestras de sangre, secreciones respiratorias, aspirados de medula ósea o tejido de piel. Estas muestras deben ser procesadas en una cabina de bioseguridad de nivel tres.

El aislamiento del hongo permite el diagnóstico definitivo de la micosis. Es un hongo de lento crecimiento, entre 15 y 21 días en su forma del moho. La confirmación del dimorfismo y el desarrollo de macroconidias a partir del microcultivo para macroconidias tuberculadas, permiten su identificación. El cultivo tiene una sensibilidad variable, del 15 al 85 %, dependiendo de la presentación clínica de la enfermedad ^33^^,^^34^.

Entre las pruebas diagnósticas no microbiológicas, se encuentran las inmunológicas para determinar antígenos o anticuerpos ^22^. La determinación de antígenos como el galactomanano, un biopolímero de *Histoplasma*, es muy útil en el diagnóstico precoz en pacientes con VIH/sida, porque influye en el tratamiento oportuno de la infección, el cual disminuye la probabilidad de mortalidad.

Actualmente, se encuentran disponibles la prueba rápida de inmunocromatografia para pruebas de flujo lateral y la prueba de inmunoadsorción enzimática (ELISA) para detectar antígenos a partir de muestras de suero, lavado broncoalveolar, líquido cefalorraquídeo y orina. La ELISA es el método más sensible, especialmente en el diagnóstico de enfermedad diseminada, con una sensibilidad del 95 % en pacientes con VIH-sida ^22^^,^^34^^-^^36^. Según la recomendación del protocolo de manejo de pacientes con HIV en Colombia, debe realizarse la prueba de antígeno de *Histoplasma* al ingreso en aquellos pacientes con un recuento de CD4+ menor de 50 por µl y según el criterio clínico ^37^.

En un estudio realizado por la Corporación de Investigaciones Biológicas de Medellín, se estandarizaron y validaron dos pruebas comerciales (MiraVista®) en muestras de orina de pacientes con HIV y sin HIV. Se determinó una sensibilidad y especificidad de la prueba de flujo lateral del 96 %, en comparación con una sensibilidad del 96 % y una especificidad del 77 % de la ELISA. La prueba de flujo lateral evaluada en este estudio tiene varias ventajas, como producir resultados en aproximadamente 40 minutos, no requerir una infraestructura compleja para la instalación del equipo, usar muestras de orina y ser fácil de realizar ^38^.

Las pruebas para detectar anticuerpos se pueden medir por las técnicas de inmunodifusión, fijación del sistema del complemento o *Western Blot*^39^. La prueba de inmunodifusión detecta los antígenos M y H de *Histoplasma*. La presencia del antígeno M indica exposición al agente patógeno y son los primeros anticuerpos en aparecer, mientras que los anticuerpos contra el antígeno H aparecen después y usualmente se relacionan con infección activa. La prueba de fijación del complemento tiene una sensibilidad del 89 % en la forma aguda, del 63 % en la diseminada en pacientes con HIV y del 98 % en la forma crónica. Si se combinan las técnicas de inmunodifusión y fijación del complemento, se observa que la sensibilidad es del 98,5 % en la enfermedad aguda, es de 67,2 % en la forma diseminada en pacientes con sida y es del 100 % en la forma crónica ^22^.

Las técnicas moleculares disponibles, utilizadas en el diagnóstico de histoplasmosis, requieren de un laboratorio de amplia complejidad, pero permiten detectar el gen *Hc100* que codifica para la proteína de 100 kDa de *Histoplasma,* a partir de muestras clínicas. Su uso en pacientes inmunocomprometidos ha adquirido relevancia debido a que reducen el tiempo de diagnóstico, lo que se traduce en una mejoría en la supervivencia de los pacientes por la rápida implementación de las terapias adecuadas ^39^. Otros diagnósticos de histoplasmosis están siendo probados, como la secuenciación de última generación ^34^.

En el protocolo de manejo de pacientes con HIV en Colombia, por considerarse zona endémica de histoplasmosis, cuando los linfocitos CD4+ están por debajo de 150 por µl, se recomienda el uso de itraconazol como profilaxis primaria ^37^.

El tratamiento de la enfermedad se basa fundamentalmente en la aplicación de anfotericina B y antimicóticos del grupo de los azoles, idealmente itraconazol, y depende de la presentación clínica del paciente: en la histoplasmosis grave o moderadamente grave, se recomienda administrar anfotericina B liposómica en dosis de 3,0 a 5,0 mg/kg/día durante dos semanas; cuando no se cuenta con esta, se recomienda administrar desoxicolato de anfotericina B en dosis de 0,7 a 1,0 mg/kg/día durante dos semanas, adoptando medidas para prevenir lesión renal. Se debe tener en cuenta que la afectación del sistema nervioso central puede requerir que se prolongue el tratamiento inicial o se aumente la dosis. Posteriormente, se administran 200 mg de itraconazol, dos veces al día, durante 12 meses. En la histoplasmosis leve a moderada, se utiliza el itraconazol en dosis de 200 mg, tres veces al día durante tres días, y luego, 200 mg dos veces al día. En los pacientes con HIV, se deja como profilaxis secundaria hasta la recuperación de los linfocitos CD4+ ^40^.

Para la prevención de la histoplasmosis, los CDC recomiendan diferentes medidas, como evitar el anidamiento o asentamiento de aves o murciélagos en las tomas de aire de los sistemas de ventilación y los procesos pulverulentos; limpiar y desinfectar locales o zonas contaminadas (por ejemplo, rociar una solución del 3 al 5 % de formalina o lejía, antes de empezar la limpieza de gallineros o zonas contaminadas) y usar elementos de protección en el caso de las personas expuestas, como el respirador aprobado por el *National Institute for Occupational Safety & Health* (NIOSH) de los Estados Unidos ^41^^,^^42^.

## Conclusiones

Para un diagnóstico temprano de la histoplasmosis, es importante hacer una anamnesis clínica completa, en la que se indague por actividades que puedan representar riesgo de exposición al hongo. Como es una infección oportunista, debe sospecharse en todo paciente inmunocomprometido, principalmente en aquellos con HIV que presenten síntomas constitucionales, tos y lesiones cutáneas.

Las instituciones de salud deben disponer de las pruebas inmunológicas para el diagnóstico de histoplasmosis mediante la detección de *H. capsulatum*, pruebas que ya se encuentran disponibles en el país. Además, independientemente de su presentación clínica, se deben documentar los casos debido a que se cuenta con escasa información epidemiológica local.

Para esto, el Instituto Nacional de Salud tiene implementada la notificación pasiva por laboratorio. En este sentido, se recomienda a los profesionales de la salud y a las entidades de salud pública, profundizar y analizar la problemática sobre la epidemiología de esta micosis y sus riesgos para la salud pública.
